# Study of dynamical heterogeneities in colloidal nanoclay suspensions approaching dynamical arrest

**DOI:** 10.1038/s41598-017-08495-9

**Published:** 2017-08-14

**Authors:** Paramesh Gadige, Debasish Saha, Sanjay Kumar Behera, Ranjini Bandyopadhyay

**Affiliations:** Soft Condensed Matter Group, Raman Research Institute, C. V. Raman Avenue, Sadashivanagar, Bangalore 560 080 India

## Abstract

The dynamics of aqueous Laponite clay suspensions slow down with increasing sample waiting time (*t*_*w*_). This behavior, and the material fragility that results, closely resemble the dynamical slowdown in fragile supercooled liquids with decreasing temperature, and are typically ascribed to the increasing sizes of distinct dynamical heterogeneities in the sample. In this article, we characterize the dynamical heterogeneities in Laponite suspensions by invoking the three-point dynamic susceptibility formalism. The average time-dependent two-point intensity autocorrelation and its sensitivity to *t*_*w*_ are probed in dynamic light scattering experiments. Distributions of relaxation time scales, deduced from the Kohlrausch-Williams-Watts equation, are seen to widen with increasing *t*_*w*_. The calculated three-point dynamic susceptibility of Laponite suspensions exhibits a peak, with the peak height increasing with evolving *t*_*w*_ at fixed volume fraction or with increasing volume fraction at fixed *t*_*w*_, thereby signifying the slowdown of the sample dynamics. The number of dynamically correlated particles, calculated from the peak-height, is seen to initially increase rapidly with increasing *t*_*w*_, before eventually slowing down close to the non-ergodic transition point. This observation is in agreement with published reports on supercooled liquids and hard sphere colloidal suspensions and offers a unique insight into the colloidal glass transition of Laponite suspensions.

## Introduction

Glasses are characterized by amorphous order and solid-like rigidities and have a wide range of applications in our day-to-day lives^1, 2^. Supercooled liquids and slightly polydisperse colloidal suspensions form glasses as the relevant control parameters, temperature (*T*) and volume fraction (*ϕ*) respectively, are changed appropriately^2, 3^. Their dynamics slow down dramatically with decreasing *T* or with increasing *ϕ* and their viscosities (*η*) or structural relaxation timescales (*τ*_*α*_) grow by several orders of magnitude. Below the glass transition temperature *T*_*g*_ or above the glass transition volume fraction *ϕ*_*g*_ (*ϕ*_*g*_ = 0.58 for hard-sphere colloidal suspensions), *η* becomes so large that no flow or structural relaxation can be detected in the system. Glass-forming liquids are called strong if *τ*_*α*_ (or *η*) shows a nearly Arrhenius growth as a function of the relevant control parameter, whereas they are termed fragile if a super-Arrhenius trend is followed^4, 5^. In many theories and phenomenological models, the dynamical slowdown and the fragile behavior of glass-forming liquids are understood by considering the cooperative movement of the constituent molecules or particles of the glass-forming liquid^6, 7^. The distinct regions exhibiting correlated particle motion are called dynamical heterogeneities (DHs). The sizes of the DHs, or alternatively, the number of molecules or particles showing correlated movement (*N*_*corr*_), grow as the glass transition is approached. The glass transition is manifested by a remarkable slowdown in the dynamics which results in the observed increase in *τ*_*α*_. Simulations and experiments have established the presence of DHs^8–10^ and the growth of *N*_*corr*_ in glass-forming liquids through the calculation of multipoint correlation functions^6, 11–17^. Experimentally, such measurements in supercooled liquids are typically performed using non-linear dielectric susceptibility^18–21^.

The present work characterises the DHs in a fragile colloidal glass. Colloidal clay suspensions are known to exhibit rich phase behavior, showing fluid, gel, ordered and disordered phases^22–25^, and serve as excellent model systems to mimic the behaviour of atomic systems^26^. One ubiquitous example of a model colloidal system is Laponite, a synthetic smectite clay which has been studied extensively for its soft glassy rheology^27^ and interesting aging properties as it spontaneously transforms from a liquid to a glass with increasing time since sample preparation, the waiting time *t*_*w*_^28–31^. In this article, we characterize the growth of *N*_*corr*_ in synthetic Laponite clay suspensions at several *t*_*w*_. Aqueous suspensions of Laponite clay are known to form Wigner repulsive glasses^23, 29, 32, 33^ in the concentration range 2–3.5 wt.% due to the buildup of long range inter-particle electrostatic repulsive interactions with increasing *t*_*w*_^28, 29^. The growth of the *α–* relaxation time in a super-Arrhenius manner as *t*_*w*_ increases has been established experimentally^28, 34, 35^ in Laponite suspensions. Recent studies in our group have established that the slowdown in the dynamics of Laponite suspensions resembles the slowdown reported in fragile glass-forming molecular liquids if *t*_*w*_ of the former system is mapped to (1/*T*) of the latter in the Arrhenius and the Vogel-Fulcher-Tammann equations, which represent, respectively, the temperature dependences of the secondary and primary relaxation modes^31, 33, 34, 36^. In an earlier study, S. Jabbari-Farouji *et al*.^37, 38^ reported DHs in Laponite suspensions by studying the particles’ rotational and translational diffusion coefficients as a function of *t*_*w*_. It was observed that the rotational diffusion of the constituent particles slows down at a faster rate than their translational motion. C. Maggi *et al*. showed collective motion and their growing sizes in aging Laponite suspensions using a time-resolved light scattering technique^39^ which combines homodyne and heterodyne dynamic light scattering methods to extract the four-point susceptibility. In these experiments, the concentration of Laponite is 1.1 wt.% where Laponite is expected to form a gel^23^.

In this study, we employ the three-point correlation function formalism^15–17^ to probe DHs and *N*_*corr*_ in Laponite suspensions of concentrations 3.0 wt.% and 3.25 wt.% with increasing *t*_*w*_ as well as by studying several concentrations between 2.0 to 3.5 wt.% (the corresponding volume fractions *ϕ* = 0.008 to 0.014) at fixed *t*_*w*_. For all Laponite suspensions studied here, a minute volume fraction of 100 nm diameter polystyrene beads are added as probe particles in dynamic light scattering experiments. For the concentration range of Laponite suspensions chosen in the present experiments, the suspensions are expected to form a repulsive glass^23^. The relaxation dynamics of Laponite suspensions are studied by analysing the intensity autocorrelation functions obtained in homodyne dynamic light scattering experiments as a function of the waiting time of the Laponite suspension *t*_*w*_ at fixed *ϕ* and also by varying *ϕ* at fixed *t*_*w*_. The scattering function decay curves are fitted by parametrizing the two-step relaxation equation (showing exponential and stretched exponential decays and representing, respectively, the secondary and primary relaxation processes of the Laponite suspension) with the control parameters *t*_*w*_ and *ϕ*. The three-point correlation functions, which are the *t*_*w*_ or *ϕ* derivatives of the decaying scattering function curves, are estimated in this analysis and are seen to exhibit peaks whose heights increase with increasing *t*_*w*_ or *ϕ*. *N*_*corr*_ is calculated from the peak height data and its evolution with <*τ*_*ww*_>/*τ*_*g*_, where *τ*_*g*_ is the relaxation time at which the Laponite suspension enters the non-ergodic state, bears a striking resemblance to existing calculations for the growth of correlations in rapidly quenched supercooled liquids. We believe that our study, which demonstrates the existence of remarkable similarities between the kinetic arrest pheonemena in Laponite clay suspensions and in supercooled liquids, provides valuable insight into the colloidal glass transition of Laponite suspensions.

## Results and Discussion

### Characterising the structural relaxation process

The intensity auto correlation function *g* ^(2)^(*q*, *t*) in DLS^40^ is related to the self-intermediate scattering function *F*_*s*_(*q*, *t*) by the Siegert relation^41, 42^,

1 $${g}^{\mathrm{(2)}}(q,t)-1=A{|{F}_{s}(q,t)|}^{2}$$

where

2 $${F}_{s}(q,t)=\frac{1}{N}\langle \sum _{j}\exp \{iq\mathrm{.[}{r}_{j}(t)-{r}_{j}\mathrm{(0)]\}}\rangle $$

**Figure 1:**
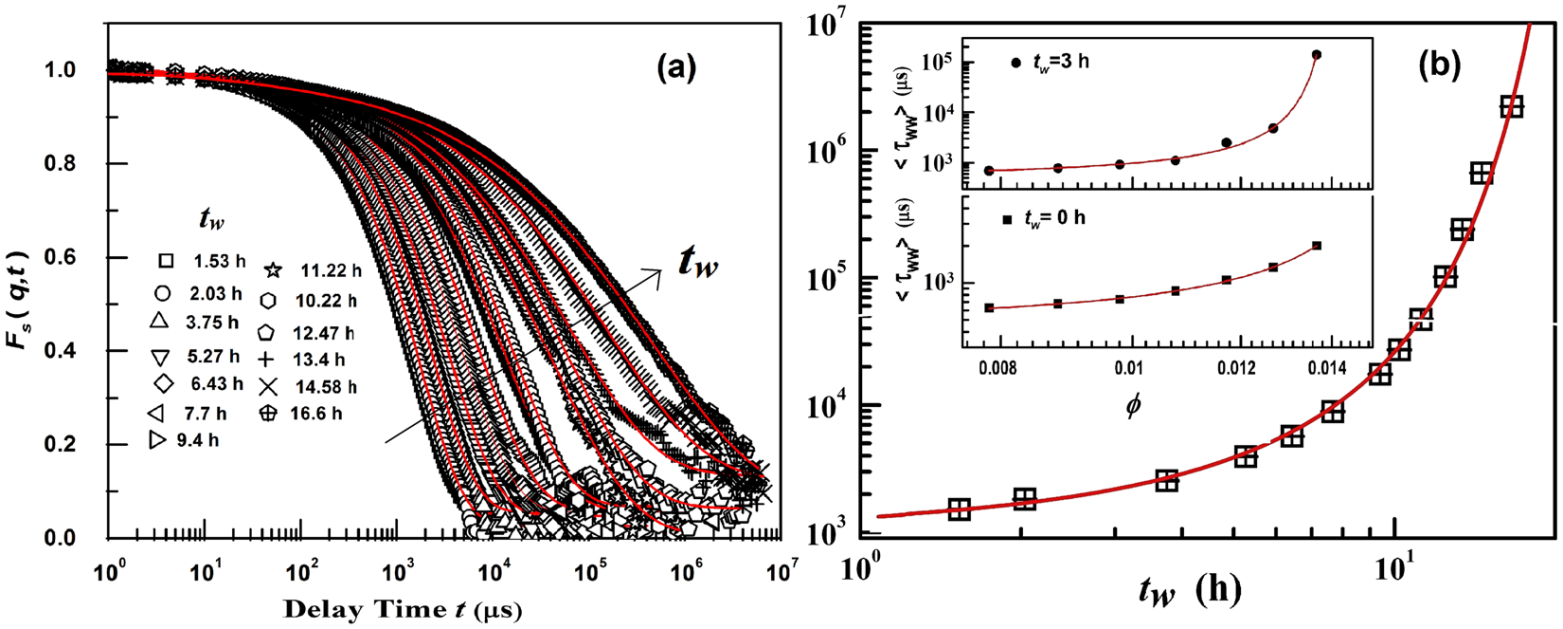
(**a**) Self intermediate intensity scattering functions (*F*_*s*_(*q*, *t*) decay curves) vs. delay times recorded at various waiting times (*t*_*w*_) at *θ* = 90° (*q* = 2.2 × 10^−2^ nm^−1^). Solid lines are fits to the two step relaxation function given in Eq. 3 and the fitting parameters obtained with increasing *t*_*w*_ are given in the Supporting Information, Table S1. (**b**) Mean structural relaxation time (<*τ*_*ww*_>) as a function of *t*_*w*_ for a Laponite-PS suspension of concentration *C*_*L*_ = 3.0 wt.%, and PS volume fraction *ϕ*_*PS*_ = 5.66 × 10^−5^. The solid line is a fit to Eq. 4. The inset in Fig. 1(b) shows the plots of <*τ*_*ww*_> vs.*ϕ* at fixed *t*_*w*_ = 0 h (bottom) and 3 h (top). Solid lines are fits to the VFT (Eq. 5) with *ϕ* as control parameter.

Here, *r*_*j*_(*t*) is the position of particle *j* at time *t*, *A* is the spatial coherence factor and the brackets represent an ensemble average. *F*_*s*_(*q*, *t*) or the two-point correlation function quantifies the relaxation dynamics between delay times of 0 and *t* seconds. *F*_*s*_(*q*, *t*) *vs*. delay time plots are obtained using DLS experiments for aging Laponite clay suspensions at several waiting times *t*_*w*_ (Fig. 1(a)). Laponite particles are extremely weak scatterers of light. Since the motivation of this work is to calculate higher order correlation functions, we have ensured a high signal to noise ratio in our measurements by homogeneously dispersing a minute quantity of PS beads (*ϕ*_*PS*_ = 5.66 × 10^−5^) in the Laponite suspensions. PS beads have a high dielectric constant mismatch with the solvent, thereby ensuring a strong scattered intensity signal^43^. Furthermore, the low concentration of the PS beads ensures that *qR*_*IPD*_ > 1 (where *R*_*IPD*_ is the inter-particle distance and *q* = 2.2 × 10^−2^ nm^−1^ for *θ* = 90°). PS particles are stabilized by negatively charged sulfate groups. The interactions between the PS and Laponite particles therefore remain repulsive. The addition of PS particles is not expected to affect the strong repulsive interactions that develop between the Laponite particles in the suspensions.The relaxation time of a 100 nm PS particle in water medium is 220 *μ*s which is less than the characteristic structural relaxation time of 630 *μ*s at *t*_*w*_ = 0 for a 2 wt% Lapo-PS suspension. The self-diffusion of the PS beads is therefore strongly affected by the aging of the underlying Laponite suspensions. Our experiments probe the collective behavior or heterogeneous dynamics of Lapo-PS particles in aqueous suspensions. The acquired data in Fig. 1(a) corresponds to the self-intermediate scattering function of the PS particles mixed uniformly in the clay suspension. The normalized *F*_*s*_(*q*, *t*) plots in Fig. 1(a) are seen to decay comparatively faster for smaller *t*_*w*_ values. As *t*_*w*_ increases, the decay of *F*_*s*_(*q*, *t*) slows down considerably. Furthermore, the decay of *F*_*s*_(*q*, *t*) can be described as a two-step process^34^ comprising a fast decaying exponential part and a comparatively slower stretched exponential part^31, 35, 36, 44^ and can be expressed as

3 $${F}_{s}(q,t)=a\,\exp \{-t/{\tau }_{1}\}+\mathrm{(1}-a)\exp \{-{(t/{\tau }_{ww})}^{\beta }\}$$

Here, *τ*_1_ is the fast or secondary relaxation time (corresponding to the diffusion of a Laponite particle inside the cage formed by the neighbors), *τ*_*ww*_ is the structural or primary *α*-relaxation time (representing its cooperative diffusion to a neighbouring position), *β* is a stretching exponent, and *a* is the weight factor for the faster secondary relaxation process^34^. Fits of the experimental data to Eq. 3 are shown in Fig. 1(a) for *C*_*L*_ = 3.0 wt.% and the fitting parameters *a*, *τ*_*ww*_ and *β*, obtained from the fits for several *t*_*w*_, are shown in Table S1 in Supporting Information (SI). *F*_*s*_(*q*, *t*) is also recorded as a function of Laponite concentration *C*_*L*_ = 2.0 wt.% to 3.5 wt.%, i.e. changing *ϕ*. In these experiments, the dynamics are probed at fixed *t*_*w*_ values 0 h and 3 h. The corresponding *F*_*s*_(*q*, *t*) vs. delay time curves along with fits to Eq. 3 with changing *ϕ* are given in SI (Fig. S1) at *t*_*w*_ = 0 and 3 h. Fitting parameters are listed in Table S2 (*t*_*w*_ = 0 h) and Table S3 (*t*_*w*_ = 3 h) with varying *ϕ*. We find good fits when *τ*_1_, corresponding to the diffusion of a single Laponite particle, is kept fixed at 30 *μ*s^34^. It was reported for glassy Laponite suspensions that the fast relaxation time *τ*_1_ (=30 *μ*s) is close to the diffusive motion of individual Laponite particles according to the Stokes-Einstein relation^34^. The dynamics probed by PS particles in clay suspensions can therefore be attributed to Laponite particle dynamics. This feature is discussed in detail in Supporting Information and ensures that the PS particles do not, in any way, interfere with the cage formation dynamics of the Laponite particles and the structural relaxation process. The growth in the structural relaxation time *τ*_*ww*_ arises from an evolution of the screened interparticle electrostatic repulsion due to a gradual process of tactoid exfoliation^45^. The dependence of the mean structural relaxation time <*τ*_*ww*_> = (*τ*_*ww*_ */β*)(Γ(1/*β*))^46^ on *t*_*w*_ is obtained from the fits and is plotted in Fig. 1(b). The *ϕ* dependence of <*τ*_*ww*_> at fixed *t*_*w*_ = 0 and 3 h are depicted in the insets of Fig. 1(b). Fragility^4, 5^ of the colloidal glasses is studied by the modified Vogel-Fultcher-Tammann (VFT) equation in which 1/*T* is replaced by *ϕ* (the relevant control parameter for colloidal glasses)^47^. Since Laponite suspensions transform to a non-ergodic state as *t*_*w*_ increases, therefore *t*_*w*_ has been used as the control parameter to study the Laponite glassy dynamics^31, 33–36, 44, 48^. The fragile behavior of Laponite suspensions is studied by employing the modified VFT equation in which *ϕ* is replaced with *t*_*w*_^34, 36^ such that the modified VFT equation can be written as follows^33, 34, 36^:

4 $$\langle {\tau }_{ww}\rangle ={\tau }_{o}\exp (\frac{D{t}_{w}}{{t}_{{\rm{\infty }}}-{t}_{w}})$$

In this equation, *τ*_*o*_ = *τ*_*α*_(*t*_*w*_ → 0), *D* is the fragility parameter (the inverse of *D* quantifies the apparent deviation from the Arrhenius trend), and *t*_∞_ is the waiting time at which the relaxation time diverges. The above equation is fitted to the data shown in Fig. 1(b) with fitting parameters, *τ*_*o*_ = 1060 ± 65 *μ*s, *D* = 6.8 ± 0.5 and *t*_∞_ = 31.3 ± 1.3 h. Such a VFT like growth of <*τ*_*ww*_> with increasing *t*_*w*_ as the Laponite suspension approaches eventual kinetic arrest is strongly reminiscent of the observations reported in fragile molecular supercooled liquids with decreasing temperature *T* and in many hard sphere colloidal glasses with increasing volume fraction *ϕ* ^17^. The VFT equation where *ϕ* is the control parameter is given below

5 $$\langle {\tau }_{ww}\rangle ={\tau }_{o}\exp (\frac{D\varphi }{{\varphi }_{\infty }-\varphi })$$

where *τ*_*o*_ = *τ*_*α*_(*ϕ* → 0) and *ϕ*_∞_ is the volume fraction at which the relaxation time diverges. The plots of <*τ*_*ww*_> vs.*ϕ*, along with fits to Eq. 5, are shown in the insets of Fig. 1(b) at *t*_*w*_ = 0 h and 3 h. The fitting parameter are *τ*_*o*_ = 395 ± 22 *μ*s, *D* = 0.57 ± 0.02, *ϕ*_∞_ = 0.01852 ± 0.0002 at *t*_*w*_ = 0 and *τ*_*o*_ = 471 ± 50 *μ*s, *D* = 0.33 ± 0.02 at *t*_*w*_ = 3 h, *ϕ*_∞_ = 0.01442 ± 0.0002. It is seen that *ϕ*_∞_ decreases with increasing *t*_*w*_. Low concentration Laponite suspensions are expected to take a longer time to reach the arrested state i.e they will have large *t*_∞_. In this work, aging dynamics are probed in detail at two different concentrations, *C*_*L*_ = 3.0 and 3.25 wt.% at several *t*_*w*_. The *F*_*s*_(*q*, *t*) decay curves and fits to Eq. 3 for *C*_*L*_ = 3.25 wt.% are shown in SI (Fig. S2(a)). The fitting parameters for a fit to Eq. 4 are *τ*_*o*_ = 1212 ± 30 *μ*s, *D* = 8.02 ± 0.2 and *t*_∞_ = 30.0 ± 0.5 h for *C*_*L*_ = 3.25 wt.%. The data is shown in Fig. S2(b).

**Figure 2:**
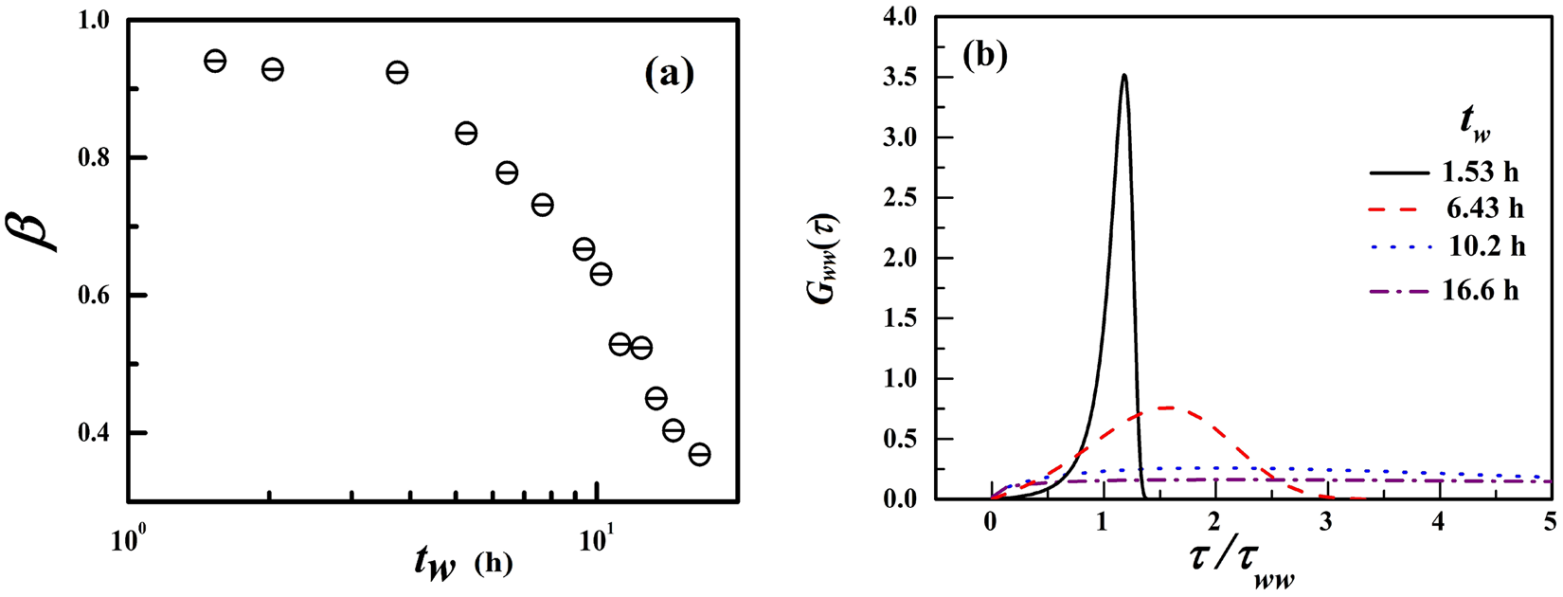
(**a**) The stretching exponent *β* vs. *t*_*w*_ and (**b**) the distribution of relaxation time scales *G*_*ww*_(*τ*) plotted at various *t*_*w*_ for the Laponite-PS system *C*_*L*_ = 3.0 wt.% of Fig. 1.

Typically, the fragile behavior of glass-forming liquids as they approach the glass transition is rationalized by enumerating the number of particles engaged in slow correlated motion, *N*_*corr*_, in the DHs and the growing sizes of these regions of cooperative motion. The *β* values, obtained from Eq. 3, are plotted as a function of *t*_*w*_ and shown in Fig. 2(a). The decrease in *β* implies that the width of time scale distributions characterising the dynamics of independently relaxing DHs increases monotonically with increasing *t*_*w*_. The correlated dynamics can therefore be modelled by assuming a linear superposition of exponential relaxation processes of the DHs, with each DH having its own relaxation time *τ* ^15^. The distribution functions of the relaxation times (*G*_*ww*_(*τ*)) with increasing *t*_*w*_ of the Laponite suspensions are calculated using the following Kohlrausch-Williams-Watts equation^46^

6 $${\rho }_{ww}(\tau )=-\frac{{\tau }_{ww}}{\pi {\tau }^{2}}\sum _{k\mathrm{=0}}^{\infty }\frac{{(-\mathrm{1)}}^{k}}{k!}\,\sin (\pi \beta k){\rm{\Gamma }}(\beta k+\mathrm{1)(}\frac{\tau }{{\tau }_{ww}}{)}^{(\beta k+\mathrm{1)}}$$

The distribution *G*_*ww*_(*τ*) = *τρ*_*ww*_(*τ*) at *t*_*w*_ = 1.53, 6.43, 10.2 and 16.6 h are plotted in Fig. 2(b) for *C*_*L*_ = 3.0 wt.%. A Laponite suspension lying in the liquid-like regime (*i*.*e*. at small *t*_*w*_ values) is characterised by a *G*_*ww*_ which shows a sharp peak at *τ*/*τ*_*ww*_ close to 1. In contrast, as the sample ages towards a non-ergodic state, a broad distribution of *G*_*ww*_ having peak position at *τ/ τ*_*ww*_ > 1 is observed. This suggests that at small sample ages, the relaxation timescales characterising the reorganization dynamics is more likely to have values within a narrow range. The broad distribution at longer *t*_*w*_ can be rationalized by considering the presence of simultaneous fast and slow moving cooperatively rearranging regions having their own independent relaxation times. The broadening of *G*_*ww*_ with increase in the control parameter *t*_*w*_ is reminiscent of observations in fragile supercooled liquids with decreasing *T* ^12, 14^ and strongly indicates the presence of heterogeneous dynamics in aging colloidal Laponite suspensions.

### Characterizing dynamical heterogeneities by evaluating three-point correlation functions

Characterizing *N*_*corr*_ of DHs in experiments is a challenging task. Theoretically, information about the sizes of the DHs and *N*_*corr*_ are embedded in the four point dynamic susceptibility which takes into account correlations in both space and time. A detailed theoretical and mathematical treatment of the four point correlation function and its relation to *N*_*corr*_ can be found in the literature^49, 50^. The four-point dynamic susceptibility *χ*_4_(*q*, *t*) is related to the fluctuating part of the self-intermediate scattering function *δF*_*s*_(*q*, *t*) by the following equation:

7 $${\chi }_{4}(q,t)={N}_{corr}\langle \delta {F}_{s}{(q,t)}^{2}\rangle $$

Experimentally therefore, *χ*_4_ can be obtained by resolving the dynamical behavior of the observable in both space and time. In contrast to molecular systems, colloidal particles have comparatively larger sizes (10 *n*m–1 *μ*m). This feature, and the easy tunability of inter-particle interactions, make colloidal systems robust model candidates for experimental and simulation studies of a variety of physical phenomena. Indeed, *χ*_4_ has been extracted for colloidal glasses using advanced microscopy techniques and simulations^6, 13, 51^. Maggi *et al*.^39^ established the growth of the four-point dynamic susceptibility (extracted from a combined homodyne and heterodyne DLS technique) as aging progresses in a low concentration (1.1 wt.%) Laponite suspension which is known to form a gel over a long time^23^. Since computation of spatial correlations from experimental data for molecular and nanocolloidal suspensions, *eg*. Laponite, is difficult, therefore in order to study DHs, a three-point correlation function, which is the lower bound of the four-point correlation function, has been introduced^18, 52, 53^. It can be accessed experimentally by probing the sensitivity of the two-point correlation function (dielectric response or the scattering function) to external control parameters such as *T* in supercooled liquids and *ϕ* in colloidal systems. For hard sphere colloids, *χ*_4_(*q*, *t*) is written as^17, 18^

8 $${\chi }_{4}(q,t)={{\chi }_{4}(q,t)|}_{\varphi }+\rho {k}_{B}T{k}_{T}{[\varphi {\chi }_{\varphi }(q,t)]}^{2}$$

where *χ*_4_(*q*, *t*)|_*ϕ*_ is the fixed density value of *χ*_4_(*q*, *t*), *ρ* is the particle number density, *k*_*T*_ is the isothermal compressibility and *χ*_*ϕ*_(*q*, *t*) is the derivative of *F*_*s*_(*τ*_*α*_, *β*) with respect to *ϕ*. The second term in Eq. 8 is the three-point dynamic susceptibility and can be accessed experimentally. The derivative of *F*_*s*_(*τ*_*α*_, *β*) with respect to *ϕ*, *χ*_*ϕ*_(*q*, *t*), has been obtained in dynamic light scattering experiments for colloidal systems^17^, while in supercooled liquids, the thermal derivative of the dielectric response *χ*_*T*_ has been used to study *N*_*corr*_^15^. In these experiments, *χ*_*ϕ*_(*q*, *t*) and *χ*_*T*_ were evaluated by probing, respectively, the scattering function and dielectric response at infinitesimal regular intervals of *ϕ* and *T*. The *ϕ*-dependence of *F*_*s*_(*q*, *t*) or the *T* dependence of the dielectric spectrum were fitted with polynomial functions. The derivatives of these fitted curves with respect to the control parameter yield the three-point susceptibilities. The use of the three point correlation function formalism to calculate *N*_*corr*_ in a wide range of glass forming liquids using dielectric and light scattering data has been successfully demonstrated by Dalle-Ferrier *et al*.^15, 16^. The authors showed that *χ*_*ϕ*_(*q*, *t*) and *χ*_*T*_ show peaks similar to *χ*_4_, with the peak heights proportional to *N*_*corr*_.

**Figure 3:**
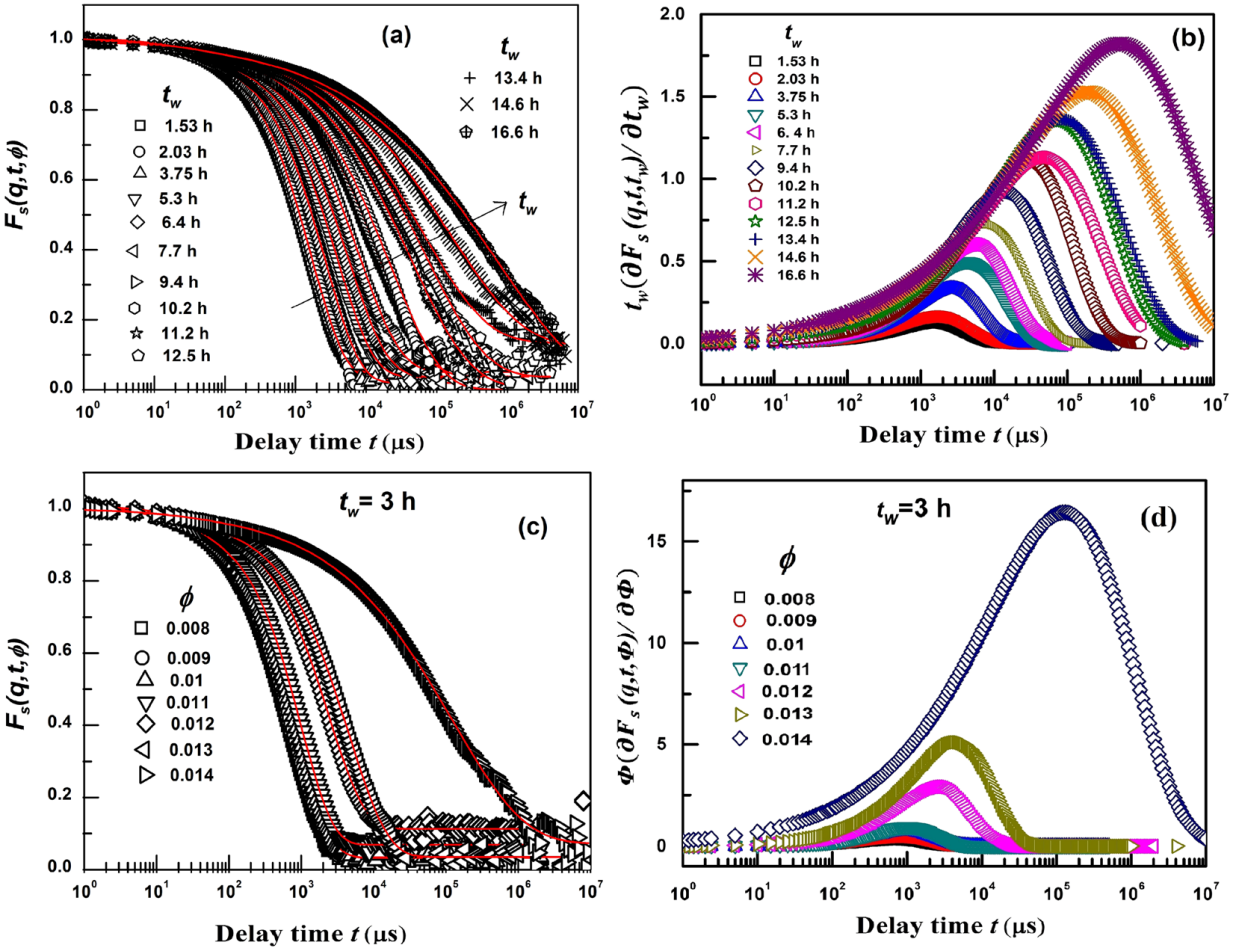
(**a**) *F*_*s*_(*q*, *t*, *t*_*w*_) decay curves at various *t*_*w*_ for Laponite-PS suspensions (Laponite concentration *C*_*L*_ = 3.0 wt.%, and PS volume fraction *ϕ*_*PS*_ = 5.66 × 10^−5^). The solid lines are fits to Eq. 9. (**b**) Plots of ${\chi }_{{t}_{w}}$(*q*, *t*) = *t*_*w*_∂*F*_*s*_(*q*, *t*, *t*_*w*_)/∂*t*_*w*_ as a function of delay time *t* for curves shown in Fig. 3(a). (**c**) *F*_*s*_(*q*, *t*, *ϕ*) decay curves at various *ϕ* at *t*_*w*_ = 3 h. The solid lines are fits to Eq. 10. (d) Plots of *χ*_*ϕ*_(*q*, *t*) = *ϕ*∂*F*_*s*_(*q*, *t*, *ϕ*)/∂*ϕ* as a function of delay time *t* for curves shown in Fig. 3(c).

In the present study, the procedure outlined above is implemented to obtain *N*_*corr*_ for aging Laponite colloidal suspensions at concentrations where they are expected to form a repulsive glass. In this regime, *t*_*w*_ is expected to behave as the control parameter for slowdown of the dynamics and is analogous to *ϕ* for hard sphere colloids^34, 35, 44^. We also compare the results of Laponite dynamics with increasing *ϕ*, the usual control parameter for colloidal glasses at fixed *t*_*w*_. Our studies involve the computation of the sensitivity of *F*_*s*_(*q*, *t*) with respect to changes in *t*_*w*_ and *ϕ* to extract the three-point correlation functions. The dependence of *F*_*s*_(*q*, *t*) on *t*_*w*_ is shown in Fig. 3(a) for *C*_*L*_ = 3.0wt.% and in Fig. 3(c) while varying *ϕ* at *t*_*w*_ = 3 h. The derivatives of *F*_*s*_(*q*, *t*) with respect to *t*_*w*_ and *ϕ* are obtained by parametrizing Eq. 3 with Eq. 4 to study aging by varying *t*_*w*_. Furthermore to analyse the data at several *ϕ* at fixed *t*_*w*_ = 0 and 3 h, Eq. 3 is parametrized with Eq. 5. The equations are given by

9 $$\begin{array}{l}{F}_{s}(q,t,{t}_{w})=a\,\exp \{-t/{\tau }_{1}\}+\mathrm{(1}-a)\exp \{-{(t/\{{\tau }_{o}\exp (\frac{D{t}_{w}}{{t}_{\infty }-{t}_{w}})\})}^{\beta }\}\end{array}$$

10 $$\begin{array}{l}{F}_{s}(q,t,\varphi )=a\,\exp \{-t/{\tau }_{1}\}+\mathrm{(1}-a)\exp \{-{(t/\{{\tau }_{o}\exp (\frac{D\varphi }{{\varphi }_{\infty }-\varphi })\})}^{\beta }\}\end{array}$$

The *F*_*s*_(*q*, *t*, *t*_*w*_) decay curves, fitted to Eqs 9 and 10, are depicted in Fig. 3(a) using solid lines for *C*_*L*_ = 3.0 wt.% and in Fig. 3(c) with varying *ϕ* at *t*_*w*_ = 3 h, (for *t*_*w*_ = 0 h, plots are shown in Fig. S3 of SI), respectively. The fitting parameters thus obtained are in good agreement with the parameters extracted by fitting the data to Eq. 3 and are tabulated in Tables S5 and S6 of the SI. The three-point correlation functions for Laponite suspensions, ${\chi }_{{t}_{w}}(q,t)$ and *χ*_*ϕ*_(*q*, *t*), are obtained by differentiating the fitted curves shown in Fig. 3(a) with respect to *t*_*w*_, *i*.*e*.${\chi }_{{t}_{w}}(q,t)={t}_{w}\frac{\partial {F}_{s}(q,t,{t}_{w})}{\partial {t}_{w}}$ and for Fig. 3(c) with respect to *ϕ i*.*e*. *χ*_*ϕ*_(*q*, *t*) = $\varphi \frac{\partial {F}_{s}(q,t,\varphi )}{\partial \varphi }$. The calculated derivatives, shown in Fig. 3(b) and (d) with increasing *t*_*w*_ at fixed *ϕ* and with increasing *ϕ* at fixed *t*_*w*_ respectively, exhibit peaks, with the peak heights increasing and the peak positions shifting to higher delay times *t* with increasing value of the control parameters. Plots of fits to Eq. 9 and ${\chi }_{{t}_{w}}(q,t)$ curves for *C*_*L*_ = 3.25 wt% are given in Fig. S3 of SI. (*χ*_*ϕ*_(*q*, *t*) at *t*_*w*_ = 0 h curves are shown in Fig. S4).

**Figure 4:**
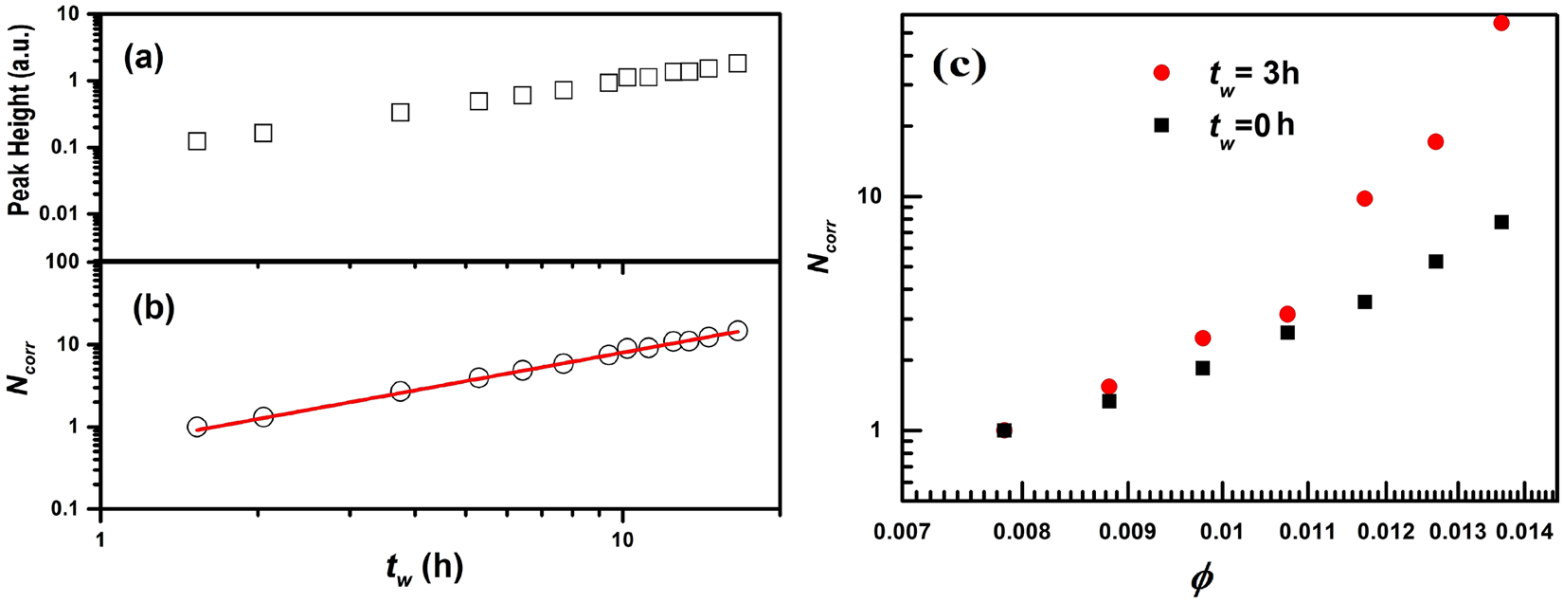
(**a**) Growth of peak height and (**b**) *N*_*corr*_ calculated from the data in Fig. 3(b) vs. *t*_*w*_ as the sample approaches the non-ergodic state. The solid line in (**b**) is a power law fit of the form *N*_*corr*_ = *B*(*t*_*w*_)^*γ*^ where *γ* = 1.17 ± 0.02 and *B* = 0.56 ± 0.04. (**c**) *N*_*corr*_ vs. *ϕ* at *t*_*w*_ = 0 and 3 h.

It has been shown that the peak heights of the three-point susceptibility in molecular liquids (control parameter *T*) and hard sphere colloids (control parameter *ϕ*) contain information of *N*_*corr*_. The peak heights of three point susceptibilities deduced in the present case with *t*_*w*_ as control parameter at fixed *ϕ* and with *ϕ* as control parameter at fixed *t*_*w*_ can therefore be expected to contain information on *N*_*corr*_ for the aging Laponite suspensions studied here. The observed growth of the peak height of ${\chi }_{{t}_{w}}(q,t)$ and *χ*_*ϕ*_(*q*, *t*), with increasing *t*_*w*_ or increasing *ϕ* establish the growing dynamical heterogeneities and the monotonically increasing trend of *N*_*corr*_ during the spontaneous aging process of Laponite colloidal suspensions. The height of the peaks, plotted in Fig. 4(a) (for *C*_*L*_ = 3.0 wt.%) as a function of *t*_*w*_, is proportional to *N*_*corr*_^15^. Next, ${N}_{corr}\propto [{t}_{w}(\frac{\partial {F}_{s}(q,t,{t}_{w})}{\partial {t}_{w}})]$ or ${N}_{corr}\propto [\varphi (\frac{\partial {F}_{s}(q,t,\varphi )}{\partial \varphi })]$ are calculated with the assumption that for the smallest waiting times or lowest *ϕ*, correlated particle motion can be ruled out (with particles moving independently, *i*.*e*. *N*_*corr*_ = 1). Here, normalization is carried out with the peak height at *t*_*w*_ = 1.5 h for *t*_*w*_ dependent study of *C*_*L*_ = 3.0 wt % or with *ϕ* = 0.008 (*C*_*L*_ = 2.0 wt %) for *ϕ* dependent data to obtain *N*_*corr*_. Since at *t*_*w*_ = 1.5 h and *ϕ* = 0.008, the stretching exponent, *β* is close to one and the distribution function of relaxation times *G*_*ww*_ peaks close to *τ*/*τ*_*ww*_ = 1 as shown in Fig. 2 (for *t*_*w*_ = 1.5 h, *β* = 0.94 ± 0.01 and *τ*/*τ*_*ww*_ = 1.18, just starting to deviate from unity). *τ*/*τ*_*ww*_ close to one suggests the presence of a unique relaxation time and the absence of correlated motion of the particles in suspension. The proportionality pre-factors in the relation for *N*_*corr*_ can be assumed to be independent of *t*_*w*_ at fixed *ϕ* as number density of Laponite particles is constant. It is to be noted here that there exists initial osmotic swelling of Laponite particles during preparation of suspensions due to hydration which is followed by build up of electrostatic repulsive interactions between the particles^23^. Therefore, over the range of aging times probed in the present experiments, the osmotic pressure and the values of *k*_*T*_ is expected to be constant. Finally, $[{t}_{w}(\frac{\partial {F}_{s}(q,t,{t}_{w})}{\partial {t}_{w}})]$ is normalized with respect to the value at *t*_*w*_ = 1.5 h at all *t*_*w*_ to obtain *N*_*corr*_ as a function of *t*_*w*_ for *C*_*L*_ = 3.0 wt.%. The corresponding *N*_*corr*_ plot is given in Fig. 4(b). *N*_*corr*_ obtained with increasing *ϕ* is shown in Fig. 4(c) at *t*_*w*_ = 0 and 3 h. We observe a small increase in *N*_*corr*_ at *t*_*w*_ = 0 h with increasing *ϕ* and obtain a large *N*_*corr*_ at high *ϕ* at higher *t*_*w*_ ( = 3 h). This is because samples of higher concentration reach the non-ergodic state at lower *t*_*w*_. Growth of peak height and *N*_*corr*_ for *C*_*L*_ = 3.25 wt.% are plotted in SI (Fig. S5) and similar trends were observed as *C*_*L*_ = 3.0 wt. %. If *τ*_*g*_ = 10 s is defined as the non-ergodic transition point^15^, a monotonic growth of *N*_*corr*_ with increasing *t*_*w*_ is observed. The growth of *N*_*corr*_ shows a power law dependence on *t*_*w*_ (*N*_*corr*_ = *B*(*t*_*w*_)^*γ*^, where the exponent *γ* = 1.17 ± 0.02, *B* = 0.56 ± 0.04) and is shown by a solid line in Fig. 4(b) for *C*_*L*_ = 3.0 wt.%. Similar power law growth of *N*_*corr*_ is observed for measurements (upto *t*_*w*_ = 9.6 *h*) performed at a lower scattering angle *θ* = 75° (*q* = 1.9×10^−2^ nm^−1^) and 60° (*q* = 1.57×10^−2^ nm^−1^) (thereby probing longer length scales 1/*q* and is displayed in the SI in Fig. S6 for *C*_*L*_ = 3.0 wt.%. Slightly higher *N*_*corr*_ values are observed at lower *q* due to the large probed length scales (45 nm to 63.6 nm for studied *q* values).

**Figure 5:**
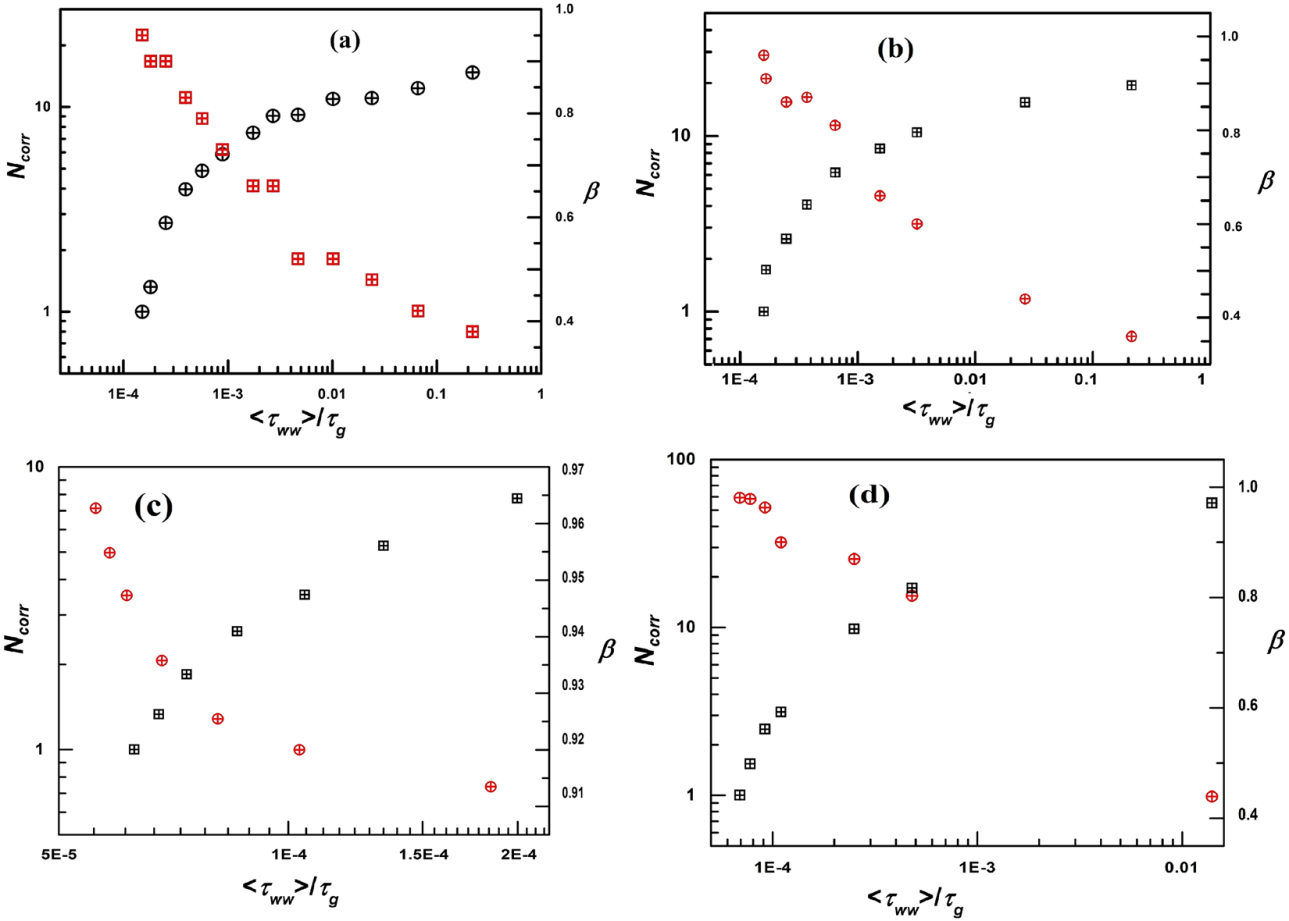
(**a**) Plot of *N*_*corr*_ (black symbols) and *β* (red symbols) *vs*. <*τ*_*ww*_>/*τ*_*g*_ for *C*_*L*_ = 3.0 wt.%, (**b**) for *C*_*L*_ = 3.25 wt.%. (**c**) with increasing *ϕ* at *t*_*w*_ = 0 h and (**d**) with increasing *ϕ* at *t*_*w*_ = 3 h. *N*_*corr*_ shows a monotonic increase, With the initial rapid increase slowing down considerably at high <*τ*_*ww*_>/*τ*_*g*_ while *β* shows a monotonic decrease.

In Fig. 5, *N*_*corr*_ is plotted as a function of *τ*_*ww*_ normalized with *τ*_*g*_ = 10 s^15^ to observe its evolution for *C*_*L*_ = 3.0 wt.% (Fig. 5(a)), 3.25 wt.% (Fig. 5(b)) and with increasing *ϕ* at *t*_*w*_ = 0 and 3 h (Fig. 5(c) and (d) respectively). *β* values are also plotted in all the figures in order to correlate the evolution of the time scale distributions with growth of *N*_*corr*_. The decrease of *β* (or increase in the width of distribution of relaxation time scales, *G*_*ww*_, as shown in Fig. 2(b)) as Laponite suspensions approach non-ergodicity indicates that the dynamics turn progressively heterogeneous.This implies growing correlations in the system, *i*.*e*. the appearance of a large number of regions of correlated groups of particles with distinct average relaxation times as the waiting time of the suspension increases. The appearance of dynamical heterogeneities of larger sizes (characterised by larger *N*_*corr*_) results in a slowdown of the dynamics which manifests as a fragile supercooled liquid-like growth of <*τ*_*ww*_> (VFT-like plot in Fig. 1(b)). However, it is seen that *N*_*corr*_ grows rapidly at small <*τ*_*ww*_>/*τ*_*g*_, but slows down considerably as the suspensions approaches the non-ergodic transition point. Remarkably, this trend is in close agreement with an observation in supercooled liquids in which *N*_*corr*_ is reported to show a power-law dependence at large *T* (*i*.*e*. in the liquid regime) and a logarithmic growth at low *T* (*i*.*e*. near the glass transition)^15, 16^. The slow increase of *N*_*corr*_ close to the non-ergodic transition is attributed to activated dynamics. In this regime, cooperative motion becomes increasingly difficult as the structural rearrangement time slows down dramatically as a consequence of the very high viscosity of the system and results in large <*τ*_*ww*_> values. Activated dynamics close to the glass transition is also seen in hard sphere glasses^17^. The increasing values of *N*_*corr*_ with *t*_*w*_, estimated from the three-point dynamic susceptibility in the present study therefore provides a quantitative measure of the fragile supercooled liquid-like heterogeneous dynamics in soft glassy colloidal Laponite suspensions.

## Conclusions

Dynamical heterogeneities in aging Laponite colloidal suspensions are investigated by the three-point dynamic susceptibility formalism with respect to the control parameters, *t*_*w*_ and *ϕ*. Laponite colloidal suspensions exhibit fragile supercooled liquid-like dynamics, with the average structural relaxation time, <*τ*_*ww*_>, in its characteristic two-step relaxation process exhibiting a VFT-like growth as a function of *t*_*w*_ at fixed *ϕ* and with varying *ϕ* at fixed *t*_*w*_. The three-point dynamic susceptibility is computed from DLS experiments by taking the derivatives of the two-point scattering decay function, *F*_*s*_(*q*, *t*), with respect to *t*_*w*_ and *ϕ*. The three-point dynamic susceptibility exhibits a peak, with the peak height growing with increasing *t*_*w*_ or *ϕ*. Our calculations show that *N*_*corr*_, the number of particles participating in correlated motion, thereby setting the size of the dynamical heterogeneity, shows a power law increase with increase in the aging time *t*_*w*_ of the Laponite suspension. Furthermore, we show that the growth of *N*_*corr*_ is initially quite fast, before slowing down close to the nonergodic transition. We believe that our study provides valuable insight into the approach of repulsive glass-forming Laponite suspensions towards kinetic arrest by demonstrating that the growth of correlations in these suspensions closely resemble the observations reported for fragile supercooled liquids^15^. Unlike in fragile supercooled liquids, however, the dynamics of Laponite suspensions are driven by athermal processes such as long-range screened inter-particle electrostatic repulsions and tactoid exfoliation. Given the similarities in the dynamical slowdown processes and the growth of correlations in Laponite suspensions and fragile supercooled liquids, the ubiquitous kinetic slowdown in glass-forming liquids, driven by a growth in heterogeneous dynamics, could well be a universal feature of fragile glass-formers.

### Sample preparation and experimental methods

Laponite RD^®^ (BYK, Inc.) powder was procured from Southern Clay products. As clay particles are hygroscopic in nature, the powder was dried in a hot oven at 120 °C for 16 h. *C*_*L*_ = 2, 2.25, 2.5, 2.75, 3.0, 3.25 and 3.5 w.t % Laponite concentration (*ϕ*_*L*_ = 1.18 × 10^−2^) suspensions were prepared by adding the dried powder slowly to Milli-Q water (resistivity 18.2 MΩ-cm). *C*_*L*_ was chosen to lie in a region of the phase space where the Laponite suspension is expected to form a Wigner glass^23, 32^. The suspension was stirred vigorously for 1 h using a magnetic stirrer. The resulting optically clear and homogeneous suspension was filtered using a 0.45 *μ*m Millipore Millex-HV grade filter using a syringe pump at a constant flow rate of 3 ml/min. A very small volume fraction (*ϕ*_*PS*_ = 5.66 × 10^−5^) of polystyrene (PS) probe particles (100 nm in diameter) was added and mixed homogeneously. The PS particles having polydispersity of 5 to 10% and stabilized with negatively charged sulfate groups are obtained from Bangs Lab (USA). These PS beads (whose sizes are less than the wavelength of the light used) are expected to act as light scatterers in the otherwise highly transparent Laponite suspensions^43^. The Laponite-PS suspensions were subsequently sealed in a cuvette. The waiting time, *t*_*w*_, was calculated from the time when the stirring of the suspension was stopped and the cuvette was sealed. Auto-correlation functions of the intensity scattered by these suspensions were recorded in dynamic light scattering (DLS) experiments^40^ using a Brookhaven Instruments Corporation BI-200SM spectrometer and a BI-9000AT digital autocorrelator. A constant temperature of 25 °C was maintained using a temperature controller (Polyscience Digital) attached to the DLS system. Details of the set-up are given elsewhere^34^. The normalized intensity autocorrelation function of the scattered light, ${g}^{\mathrm{(2)}}(q,t)=\frac{ < I(q\mathrm{,0)}I(q,t) > }{ < I(q{\mathrm{,0) > }}^{2}}$, was recorded as a function of delay time *t*. Here, *q* and *I*(*q*, *t*) are the scattering wave vector and the intensity of the scattered light at a particular *q* and *t* respectively. *q* is related to the scattering angle *θ* by the equation *q* = (4*πn*/*λ*)sin(*θ/2*), where *n* and *λ* are the refractive index of the medium (*n* = 1.334) and the wavelength of the laser (*λ* = 532 nm) respectively^40^. The intensity autocorrelation data was recorded at *θ* = 90° (q = 2.2×10^−2^ nm^−1^), *θ* = 75° (q = 1.9 × 10^−2^ nm^−1^) and *θ* = 60° (q = 1.6 × 10^−2^ nm^−1^). The three point correlation functions were computed by taking the derivatives of the time-dependent self-intermediate scattering function using Mathematica with respect to *t*_*w*_ at fixed *C*_*L*_ = 3.0 and 3.25 wt. % and with respect to *ϕ* at fixed *t*_*w*_ = 0 and 3 h for all the concentrations studied.

## Electronic supplementary material

Supplementary Informaton
