# Supplementary material for: Study of dynamical heterogeneities in colloidal nanoclay suspensions approaching dynamical arrest
Source: Sci Rep. 2017 Aug 14;7:8017. doi: 10.1038/s41598-017-08495-9 (PMC5556041; doi:10.1038/s41598-017-08495-9)
Supplement: Supplementary file 1 — Supplementary Informaton [file 41598_2017_8495_MOESM1_ESM.pdf]

Supporting Information

Study of dynamical heterogeneities in colloidal nanoclay suspensions approaching dynamical arrest

*Paramesh Gadige, Debasish Saha, Sanjay Kumar Behera, and Ranjini Bandyopadhyay**

Soft Condensed Matter Group, Raman Research Institute, C. V. Raman Avenue, Sadashivanagar, Bangalore 560080, INDIA.

*ranjini@rri.res.in

Note on determination of τ_1:

The relaxation time (τ_1) of a spherical particle is related to its diffusion coefficient D_1 by: $\tau_1 = 1/D_1 q^2$ where q is the scattering wave vector. According to the Stokes-Einstein (SE) relation, $D_1 = k_B T/6\pi\eta r_h$ for a dilute suspension of spherical monodisperse particles, where k_B, T, η and r_h are the Boltzmann constant, temperature, viscosity of the medium and hydrodynamic radius of the particle respectively. Since the Laponite platelet is a disk-shaped particle with diameter d=25-30nm and thickness h=1nm, its equivalent spherical diameter (ESD) (d_s) is given by the Jennings-Parslow relation [1]

$$d_s = d\left(\frac{3\tan^{-1}\left(\sqrt{\left(\frac{d}{h}\right)^2-1}\right)}{2\sqrt{\left(\frac{d}{h}\right)^2-1}}\right)^{1/2}$$

From the above relation, the effective spherical diameter of the Laponite particle is d_s= 7.5-8.3 nm. τ_1 is related to spherical particle radius ($r_h = d_s/2$) through the SE relation

$$\tau_1 = \frac{1}{D_1 q^2} = \frac{6\pi\eta r_h}{k_B T q^2}$$

For q = 0.0223 nm^{-1}, η=0.89 mPa.s at T=25°C, $\tau_1 \approx 30 - 34$ μs which corresponds to d_s= 7.5-8.3 nm . This τ_1 value is kept fixed while fitting the experimental data with Eq.3 and Eq.8.

Table S1: Fitting parameters obtained by fitting Eq.3 to the data shown in Fig.1(a) with increasing t_w.

$t_w(h)$	a (±0.003)	$<\tau_{ww}>=(\tau_{ww}/\beta)\Gamma(1/\beta)$ (μs)	β (±0.01)
**1.53**	0.036	1513.8±11.8	0.94
**2.03**	0.037	1820.6±13.7	0.93
**3.75**	0.061	2545.1±24.6	0.92
**5.27**	0.028	3939.5±25.3	0.83
**6.43**	0.022	5684.8±33.1	0.78
**7.7**	0.025	8886.5±61.4	0.73
**9.4**	0.022	17453.1±116.9	0.67
**10.22**	0.02	27131.7±253.4	0.63
**11.22**	0.002	46977.4±589.1	0.53
**12.47**	0.009	101532.2±594.5	0.52
**13.4**	0.007	239309.1±2877.6	0.45
**14.58**	0.018	662571. 9±3277.1	0.40
**16.6**	0.006	$2.2\text{x}10^6$±7814.6	0.37

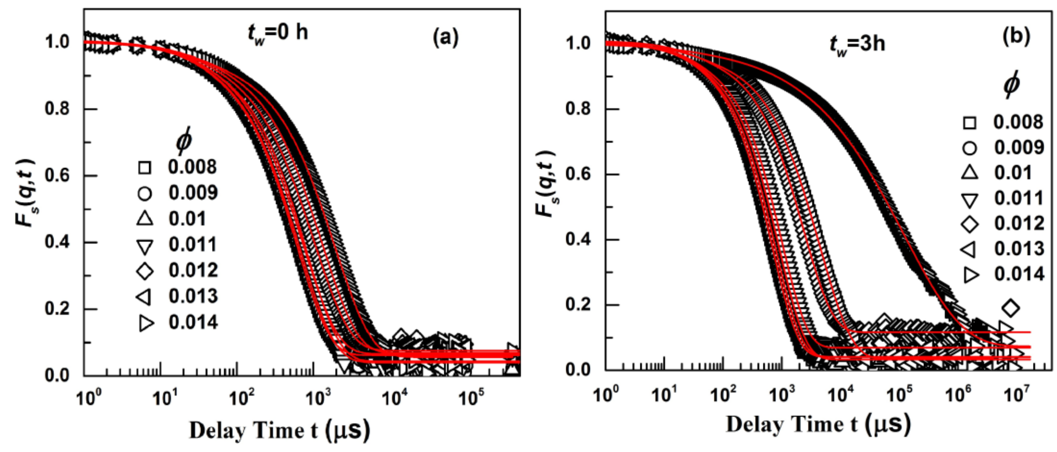

Fig.S1: Self intermediate intensity scattering functions F_s (q,t) decay curves (at θ=90^o(q= 2.2 x10^{-2} nm^{-1})) vs. delay times recorded at various volume fractions of Laponite-PS suspensions at t_w=0 and 3h. Solid lines are fits to the two step relaxation function given in Eq.3.

Table S2: Fitting parameters obtained by fitting Eq.3 to the data shown in Fig. S1 with increasing volume fraction at t_w=0

**C (wt%)**	***Φ***	***a* (±0.003)**	**$<\tau_{ww}>=(\tau_{ww}/\beta)\Gamma(1/\beta)$ (μs)**	***β* (±0.01)**
2.0	0.008	0.033	628. ±10.7	0.96
2.25	0.009	0.034	676.5±12.6	0.96
2.5	0.01	0.033	735.5±11.1	0.95
2.75	0.011	0.003	857.3±15.4	0.94
3.0	0.012	0.039	1051.1±18	0.93
3.25	0.013	0.048	1334.2±11	0.92
3.5	0.014	0.055	1997.7±20	0.91

Table S3: Fitting parameters obtained by fitting Eq.3 to the data shown in Fig. S1 with increasing volume fraction at t_w=3h

**C (wt%)**	***Φ***	***a* (±0.003)**	**$<\tau_{ww}>=(\tau_{ww}/\beta)\Gamma(1/\beta)$ (μs)**	***β* (±0.01)**
2.0	0.008	0.037	690.4±20.9	0.98
2.25	0.009	0.041	776.3±26.1	0.98
2.5	0.01	0.037	917.5±21	0.96
2.75	0.011	0.022	1098.9±12	0.9
3.0	0.012	0.043	2504.4±44	0.87
3.25	0.013	0.035	4793.7±34	0.80
3.5	0.014	0.006	139705.1±74	0.44

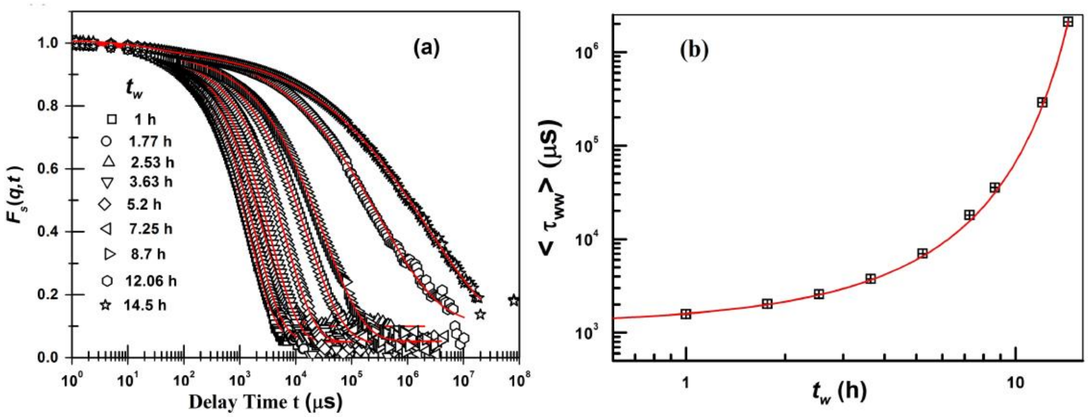

Fig. S2 (a) $F_s(q,t)$ decay curves vs. delay times recorded at various t_w at θ=90° for a Laponite-PS suspension of concentration C_L = 3.25 wt.%, and PS volume fraction Φ_{PS}= 5.66 x10^{-5}. Solid lines are fits to the two step relaxation function given in Eq.3 and the fitting parameters obtained with increasing t_w are given in the, Table S4. (b) Mean structural relaxation time < τ_{ww} > as a function of t_w The solid line is a fit to Eq.4.

Table S4: Fitting parameters obtained by fitting Eq.3 to the data shown in Fig.S. 2(a) with increasing t_w.

t_w(h)	*a* (±0.003)	<τ_{ww}>=(τ_{ww}/β)Γ(1/β) (μs)	β (±0.01)
1	0.059	1570.1±20	0.96
1.77	0.045	2018.6±18.3	0.90
2.53	0.037	2572.6±19.8	0.85
3.63	0.047	3745.9±30.6	0.86
5.23	0.05	6977. 0±41.5	0.81
7.25	0.032	18024.4 ±37.7	0.67
8.7	0.029	35563.9± 87.9	0.60
12.1	0.028	288098.7±218	0.45
14.5	0.026	2.10928E6±1441.8	0.36

Table S5: Fitting parameters obtained by fitting the data shown in Fig.3 (a) to Eq.9 (shown in main text) with increasing t_w. The input parameters in the equation are τ_1=30 μs and VFT parameters, D=6.82±0.5, t_∞=31.3±1.3 h, τ_o=1060±65 μs.

t_w(h) **(±0.2)**	***a*** **(±0.003)**	***β*** **(±0.01)**
**1.53**	0.042	0.95
**2.03**	0.02433	0.90
**3.75**	0.041	0.90
**5.3**	0.026	0.83
**6.4**	0.034	0.79
**7.7**	0.0196	0.73
**9.4**	0.019	0.66
**10.2**	0.042	0.67
**11.2**	0.01	0.52
**12.5**	0.00952	0.53
**13.4**	0.012	0.48
**14.58**	0.004	0.42
**16.6**	0.009	0.38

Table S6: Fitting parameters obtained by fitting the data shown in Fig.S4 and Fig. 3(c) to Eq.10 with increasing Φ at t_w= 0 and 3h. The input parameters in the equation are τ_1=30 μs and VFT parameters, at t_w= 0 h are D=0.57±0.02, Φ_∞=0.01852±0.0002, τ_o=395±22 μs and for t_w=3 h are D=0.33±0.02, Φ_∞=0.01442±0.0002, τ_o=471±50 μs

**C**	***Φ***	***a*** **(t_w=3h) (±0.003)**	**β (t_w=3h) (±0.01)**
2.0	0.008	0.044	0.99
2.25	0.009	0.051	0.99
2.5	0.01	0.054	0.99
2.75	0.011	0.043	0.98
3.0	0.012	0.042	0.87
3.25	0.013	0.035	0.80
3.5	0.014	0.006	0.44

C	*Φ*	***a*** **(t_w=0) (±0.003)**	**β (t_w=0h) (±0.01)**
2.0	0.008	0.056	0.99
2.25	0.009	0.034	0.96
2.5	0.01	0.053	0.98
2.75	0.011	0.039	0.99
3.0	0.012	0.056	0.95
3.25	0.013	0.065	0.97
3.5	0.014	0.059	0.92

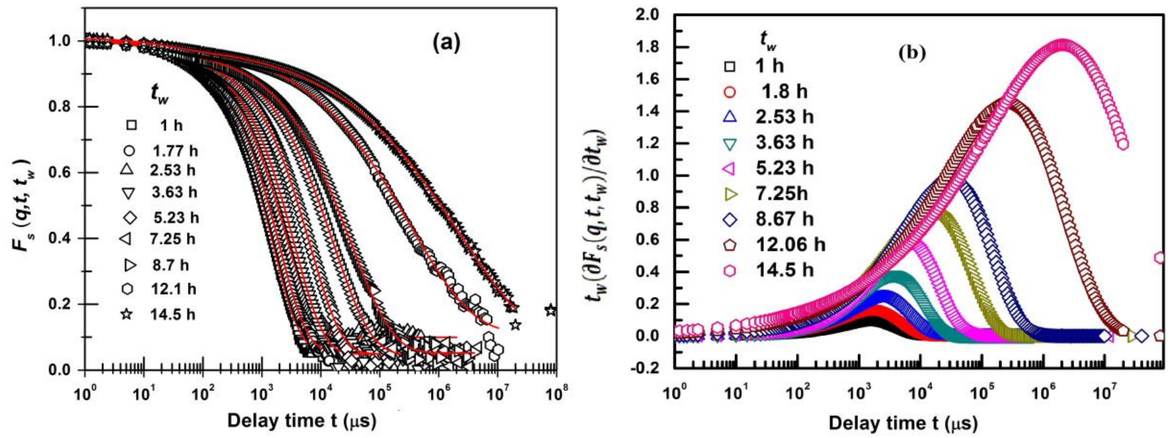

Fig.S3. $F_s(q,t)$ decay curves at various t_w for C_L = 3.25 wt.%,. The solid lines are fits to Eq.9. (b) Plots of $\chi_{tw} = t_w\, \partial F_s(t,tw)/\partial t_w$ as a function of delay time t.

Table S7: Fitting parameters obtained by fitting the data shown in Fig.S3 to Eq.9 (shown in main text) with increasing t_w. The input parameters in the equation are τ_1=30 μs and VFT parameters, D=8.02±0.2, t_∞=30.0±0.5 h, τ_o=1212±30 μs.

***$t_w(h)$* (±0.2)**	***a* (±0.003)**	***β* (±0.01)**
1	0.061	0.96
1.77	0.051	0.91
2.53	0.047	0.86
3.63	0.049	0.87
5.23	0.051	0.81
7.25	0.03	0.66
8.7	0.029	0.60
12.1	0.002	0.44
14.5	0.011	0.36

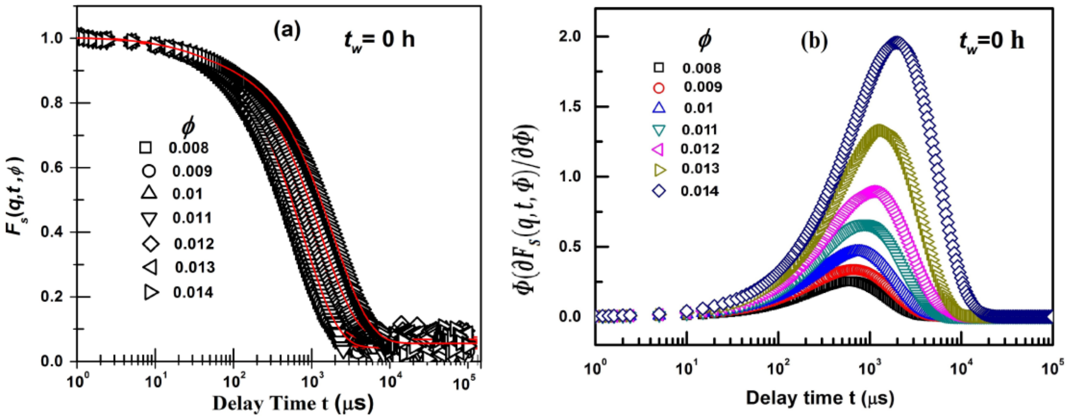

Fig.S4: (a) $F_s\,(q,t,\Phi)$ decay curves at various φ for Laponite-PS suspensions at t_w=0. The solid lines are fits to Eq.10. (b) Plots of χ_Φ(q,t)= $\varphi\partial F_s(t,\Phi)/\partial\Phi$ as a function of delay time t for the varying Laponite concentration at t_w=0.

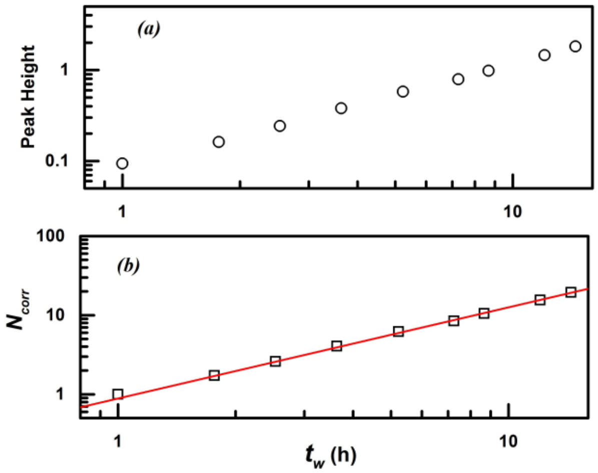

Fig.S5. (a) Growth of peak height and (b) N_{corr} calculated from the data in Fig. S3 (C_L=3.25 wt.%) as t_w approaches the non-ergodic state. The solid line is a power law fit of the form $N_{corr}=B(t_w)^{\gamma}$ γ = 1.15 and B = 0.88.

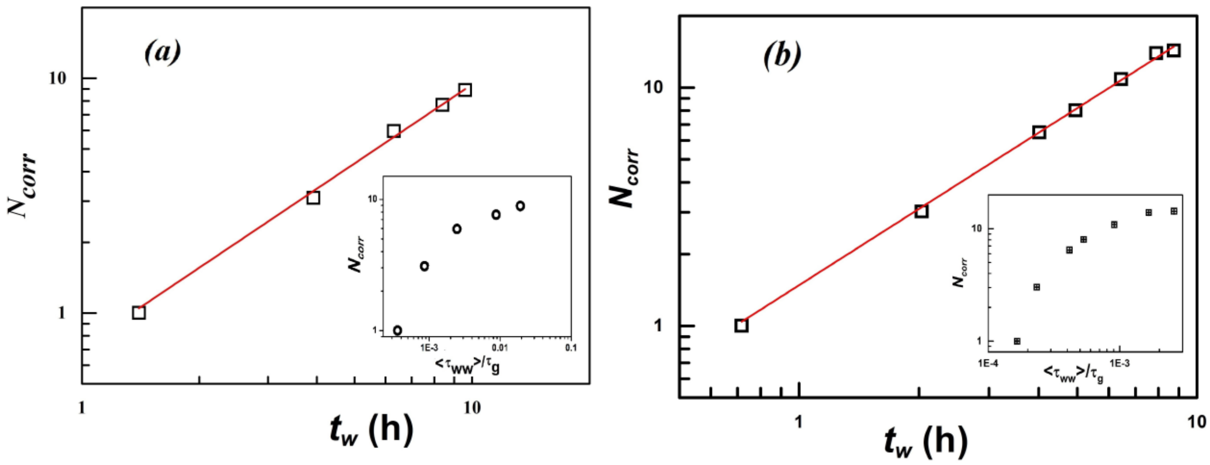

Fig.S6: (a) Growth of the number of correlated particles (N_{corr}), deduced at a scattering angle $\theta=60^{o}$ and (b) 75°, as the Laponite-PS (C_L=3.0 wt%) system approaches the non-ergodic transition. The solid line is the power law fit of the form $N_{corr}=B(t_w)^{\gamma}$ with B=0.72±0.1 and exponent γ=1.12±0.06 at $\theta=60^{o}$ and B=1.4±0.1 and exponent γ=1.06±0.06 for $\theta=75^{o}$. Inset shows the plot of N_{corr} vs. $< \tau_{ww} > /\tau_g$. N_{corr} shows a monotonic increase, with the initial rapid increase slowing down considerably at high $< \tau_{ww} > /\tau_g$.

References

1. B.R. Jennings and K. Parslow, Proc. R. Soc. London A, **419,** 137 (1988).
